# Chronic Hepatitis E Virus Manifesting as Elevated Transaminases in a Heart Transplant Patient

**DOI:** 10.14309/crj.0000000000001308

**Published:** 2024-03-22

**Authors:** Islam Mohamed, Kimberly Sanders, Donald J. Hillebrand

**Affiliations:** 1 Department of Internal Medicine, University of Missouri-Kansas City, Kansas City, MO; 2 Department of Gastroenterology and Hepatology, University of Missouri-Kansas City, Kansas City, MO; 3 Department of Gastroenterology and Hepatology, University of Kansas Health System, Kansas City, KS

**Keywords:** Hepatitis E Virus, Heart Transplantation, Immunosuppression, HEV in Solid Organ Transplant Recipients, Antiviral Therapy for HEV

## Abstract

Hepatitis E virus (HEV) is a common cause of viral hepatitis worldwide. Genotypes 1 and 2 cause acute hepatitis in endemic regions (Asia and Africa), whereas genotypes 3 and 4 (America and Europe) result in sporadic acute or chronic hepatitis, specifically in certain groups. HEV infections are rising because of increased transplantation rates and immunosuppression. We report a 75-year-old heart transplant patient with nonspecific symptoms, diagnosed with HEV chronic hepatitis. Despite ribavirin-induced hemolytic anemia, the patient achieved sustained virological response and normalization of liver enzymes.

## INTRODUCTION

Hepatitis E virus (HEV) is a common cause of acute and chronic viral hepatitis around the world. The World Health Organization estimates that there are approximately 20 million new cases of HEV yearly.^1^ HEV is often overlooked as a cause of chronic hepatitis because of its relatively low prevalence in developed nations. However, its incidence has been rising in the past decade because of the increased number of transplanted recipients and the increased use of immunosuppressants. Despite this trend, there are no established guidelines or consensus regarding the management or treatment of chronic HEV in solid organ transplant patients (SOT). We describe the case of a 75-year-old man with a history of heart transplantation in 2015, who was referred to the hepatology clinic for evaluation of elevated liver enzymes and was diagnosed with chronic HEV infection and was treated with ribavirin.

## CASE REPORT

**Figure 1.**
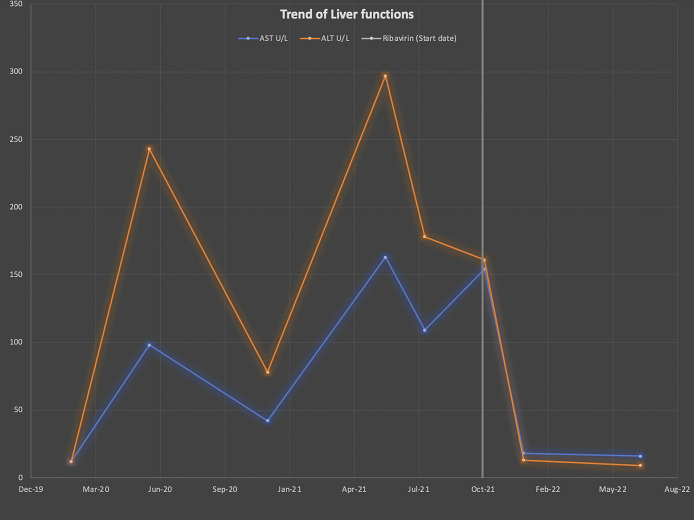
Trend in Liver Functions after starting Ribavirin treatment.

A 75-year-old man presented to the hepatology clinic for evaluation of abnormal liver enzymes in the setting of cardiac transplantation and longstanding immunosuppression. Medical history included type 2 Diabetes Mellitusc (DM), Chronic Kidney disease (CKD) 3a, hypertension, and dyslipidemia. The patient underwent a successful orthotopic heart transplant in 2015 for end-stage nonischemic cardiomyopathy. Both the recipient and donor were cytomegalovirus (CMV) IgG-positive but negative for CMV IgM. In addition, both tested negative for hepatitis A, B, and C, without any screening for HEV. His immunosuppressive regimen consisted of tacrolimus 2 mg twice daily (target level: 5–8) alongside prednisone at 5 mg daily. He had a single episode of mild acute cellular rejection 1 year after transplant but did not have donor-specific antibodies. He was first noted to have mild-moderate elevation in liver enzymes with hepatocellular pattern (Aspartate Aminotransferase (AST)/Alanine Transaminase (ALT) 100–200 s U/L) in June 2020 (Figure 1). Liver enzymes improved but remained persistently elevated prompting hepatology referral in October 2021. His symptoms included constant sharp infraumbilical abdominal pain for 1 year as well as fatigue, poor appetite, and night sweats since transplantation. Risk factors for liver disease included tattooing, exposure to blood/body fluids, and occupational chemical/toxin exposure from his work in a factory. He consumes properly cooked meats and denies consumption of game meat. Various imaging modalities (ultrasound, computed tomography scan, and magnetic resonance imaging) showed no abnormalities. Bidirectional endoscopy revealed colonic diverticulosis. Echocardiogram showed preserved ejection fraction and no evidence of cardiac dysfunction. Laboratory workup, including alpha-1-antitrypsin levels, Anti-nuclear Antibody (ANA), Anti-nuclear Antibody (ANCA), antismooth muscle antibodies, hepatitis A, B, and C, immunoglobulin levels, iron panel, antimitochondrial antibodies, celiac screening, protein electrophoresis, Cytomegalovirus (CMV) polymerase chain reaction, and Epstein-Barr virus (EBV) polymerase chain reaction, yielded no significant findings. Serologies revealed positive HEV IgM and IgG antibodies with HEV RNA quantitative of 5,600,000 IU/mL consistent with chronic HEV infection. Treatment was initiated with ribavirin 600 mg daily in the setting of CKD. He demonstrated a rapid virological response with normal liver enzymes and undetectable HEV RNA after only 4 weeks of treatment. Unfortunately, approximately 2 months after initiating treatment, he experienced ribavirin-induced hemolytic anemia, resulting in a drop in hemoglobin from 14.2 to 9 g/dL and platelet levels from 173 to 110 Th/μL. This required further reduction in dose to 400 mg daily and eventually 200 mg daily. He completed 12 weeks of ribavirin, and his hemoglobin subsequently normalized 1 month after discontinuation. Six months after treatment, his liver enzymes normalized, and HEV levels were negative, indicating a sustained virological response.

## DISCUSSION

HEV is a common cause of acute viral hepatitis worldwide, with population incidence rates up to 30% in adults.^2^ The transmission of this RNA virus is traditionally through the fecal-oral route in endemic areas (genotypes 1 and 2). In nonendemic, developed areas, transmission is primarily food-borne or by direct contact with infected animals.^2,3^ Genotypes 1 and 2 are typically associated with acute viral hepatitis, whereas genotypes 3 and 4 are more likely to result in chronicity in immunocompromised individuals. Acute HEV is self-limiting presenting as an asymptomatic elevation in liver enzymes, acute viral febrile illness, or prolonged cholestasis. Acute liver failure is uncommon (approximately 0.5%–4%), but when present, can be highly fatal, with mortality rates up to 25% in certain patient groups (pregnant women and chronic liver disease).^4,5^ The resolution of acute hepatitis or progression to chronicity is believed to be related to the host's T-cell response. Chronic HEV, defined by the presence of Polymerase chain reaction (PCR) HEV in serum or stool for at least 3–6 months, is almost exclusively limited to immunocompromised patients including HIV and SOT recipients.^2^ A recent meta-analysis found that the prevalence of HEV in SOT is ∼20% with the highest prevalence in liver transplant recipients (27.2%).^6^ Presentation is typically mild or asymptomatic with persistently elevated liver enzymes. In immunocompromised patients, liver enzymes are usually only mildly elevated compared with the high levels seen in immunocompetent individuals with acute HEV. Chronic HEV can also lead to the rapid development of fibrosis and cirrhosis, but the frequency of this complication remains unknown. The use of tacrolimus rather than cyclosporine was shown to be an independent risk factor for the development of chronic HEV, possibly because of the stronger immunosuppressive effects of tacrolimus compared with cyclosporin.^7^

Treatment of chronic HEV should first be aimed at reducing immunosuppressive therapy if feasible. According to Kamar et al, 32% (18 of 56 patients) of chronic HEV cases successfully cleared the virus with tacrolimus dose reduction alone with no evidence of recurrence or relapse observed.^7^ These findings were observed after a reduction in the daily tacrolimus dose from 0.09 ± 0.09 mg/kg/d to 0.06 ± 0.05 mg/kg/d, accompanied by a decrease in trough levels from 10.1 ± 4.3 ng/mL to 7.9 ± 3.3 ng/mL.^7^ Our decision not to reduce this patient's tacrolimus dose was based on both his previous acute cellular rejection of the cardiac allograft and his remote location from his cardiac transplant center and the difficulty of monitoring his immunosuppression remotely. Ribavirin is considered first-line therapy for those unable to reduce immunosuppression, unresponsive to immunosuppression reduction, or that have other indications for treatment of HEV.^5,7^ Unfortunately, there are no established guidelines or recommendations for the dosing or duration of ribavirin treatment. Kamar et al performed a retrospective, multicenter case series to evaluate ribavirin for the treatment of chronic HEV in transplant recipients. The median dose of ribavirin was 600 mg with a median duration of treatment of ∼3 months. Initial doses were adjusted for renal function. Results demonstrated HEV clearance in 95% of patients at the end of therapy. Recurrence was observed in 10 patients of which 6 were retreated and 4 achieved Sustained Virological Response (SVR) after a prolonged course of ribavirin. Overall, 78% (46 of 59 patients) achieved a sustained virologic response, defined as an undetectable serum HEV RNA level 6 months after completing ribavirin therapy.^8^ As expected, the most common side effect observed was anemia in which 29% required a dose reduction, 12% required blood transfusions, and 54% required erythropoietin.^8^ Patients with CKD are at higher risk of hemolysis because ribavirin is renally excreted, so close laboratory monitoring is required. Pegylated interferon alpha can be considered if ribavirin treatment fails to resolve the chronic HEV or if the patient is intolerant to ribavirin therapy but should be used with caution, given its risk of graft rejection.^9^ In a multicenter study, sofosbuvir exhibited minimal effectiveness in reducing chronic HEV viral load, with failure of viral clearance in all the patients treated.^10^ Other medications, including interferon lambda, mycophenolic acid, zinc, and ivermectin, which have shown efficacy in animal models, are yet to be studied in humans.^11^ Other medications, including interferon lambda, mycophenolic acid, zinc, and Ivermectin, which have shown efficacy in animal models, are yet to be studied in humans.^11^

## DISCLOSURES

Author contributions: I. Mohamed: writing—original draft, review and editing, and visualization. K. Sanders: writing—original draft, writing—review and editing of the manuscript. DJ Hillebrand: supervising and reviewing final draft. Dr Donald Hillebrand, is the article guarantor.

Financial disclosure: None to report.

Informed consent was obtained for this case report.
